# Challenges and opportunities for implementing digital health interventions in Nepal: A rapid review

**DOI:** 10.3389/fdgth.2022.861019

**Published:** 2022-08-25

**Authors:** Rojina Parajuli, Dipak Bohara, Malati KC, Selvanaayagam Shanmuganathan, Sabuj Kanti Mistry, Uday Narayan Yadav

**Affiliations:** ^1^ Department of Public Health, Torrens University, Sydney, Australia; ^2^ Menzies Centre for Health Policy and Economics, The University of Sydney, New South Wales, Sydney, Australia; ^3^ Centre for Primary Health Care and Equity, Faculty of Medicine,, University of New South Wales, NSW, Sydney, Australia; ^4^ Department of Public Health, Daffodil International University, Dhaka, Bangladesh; ^5^ National Centre for Epidemiology and Population Health, The Australian National University, ACT, Canberra, Australia

**Keywords:** challenges, digital health, implementation, opportunities, telemedicine, telehealth, e-health, Nepal

## Abstract

**Background:**

In recent times, digital technologies in health care have been well recognized in Nepal. It is crucial to understand what is works well and areas that need improvements in the digital health ecosystem. This rapid review was carried out to provide an overview of Nepal's challenges and opportunities for implementing digital health interventions.

**Methods:**

This study is reported according to PRISMA guidelines and used telehealth, telemedicine, e-health, mobile health, digital health, implementation, opportunities, challenges and Nepal as key search terms to identify primary studies published between 1 January 2010 and 30 December 2021 in four databases, namely PubMed, Google Scholar, Scopus, and CINAHL. Initially, identified studies were screened against predetermined selection criteria, and data were extracted, and the findings were narratively synthesized.

**Result:**

The review identified various challenges, opportunities, and benefits of implementing digital health initiatives in Nepal. The most expressed challenge was inadequate technical facilities (lack of electricity and internet) and rugged geographical distribution, which makes transportation difficult in hilly and mountain areas. Shortage of skilled workforce and supportive policies were also notable challenges documented. Meanwhile, major opportunities identified were education and training of the students and health practitioners and increasing awareness among the general population.

**Conclusion:**

This review identified various factors associated with the successful implementation of digital health initiatives in Nepal. Our findings may guide the formulation of digital health policy and interventions to improve mass health outcomes using digital health services.

## Introduction

In the 21st century, digital health can connect healthcare systems and deliver health services to promote health outcomes for people of all ages (1). The field of knowledge and practice are essentially associated with the development and use of digital technologies to improve health (2). The World Health Organization defined telemedicine as “*the delivery of health care services, where distance is a critical factor, by all health care professionals using information and communication technologies for the exchange of valid information for the diagnosis, treatment and prevention of disease and injuries, research and evaluation, and for the continuing education of health care providers, all in the interests of advancing the health of individuals and their communities*” (3). Similarly, Jacob et al. (2020) defined mobile health (mHealth) as the medicinal practice conducted through any portable gadget like a cell phone or patient monitoring device. Digital health encompasses a comprehensive approach to providing health care services to the patients in different forms like synchronous and asynchronous or through remote monitoring and mobile health (4). The advantage of deploying telehealth is enhancing the ease of access to health services (1). Most developed countries have effectively used digital health, and many developing countries follow the pattern of adopting digital health (5)

In Nepal, a lower-middle-income country situated between China and India, 83% of the population lives in rural areas. One-fourth of the population is classified as “under the poverty line” (6–8). Recent data from 2021 shows that 92.54% of the Nepalese population takes 15 min with motorized travel mode and 94.63% take 60 min of walking distance to have to access health care facilities in Nepal (9), health infrastructure is poorly developed, and the country has a low health human index (10). In rural areas, establishing well-equipped health care centers with specialized health services is an ongoing challenge for the government (10). A poor economy is an important but not the sole barrier to healthcare access in Nepal. Nepal's rugged terrain, especially in the mountains, makes transportation, and installation difficult; consequently, access to visiting health facilities is limited (7). With multiple challenges surrounding effective health care delivery, the government's plan to make universal health coverage is not fully illustrated. Therefore, digital health is considered one of the promising resources to make health care accessible cost-effectively, especially in tough-to-reach areas (5, 10).

In the context of rural Nepal, telehealth offers great opportunities such as remote consultation of medical practitioners (otherwise practicing in urban areas) (11, 12), remote delivery of specialized services, distance education and training of local health care providers, and collaboration of local health workers with other national and international experts. Such distance learning and collaboration can bring positive changes among the health care providers regarding their skills and the services they provide (4). Additionally, using information and communication technology (ICT), digital health may help to improve health literacy and bring positive change in people's health behaviours (13). Digital health also connects patients electronically with the health care providers so that personalized medical plans can be developed to deliver better health care for improved health outcomes (14)

In Nepal, the Telecommunication Act and National Telecommunication Policy were established in the late 1990s. The digital health system was introduced as HealthNet in 1995 (11, 15) by a non-governmental organization (NGO) to provide affordable internet services for people to access health care facilities. After that, in 2002 the “Nepal wireless project” and “hello-health” were established to provide ICT access and digital health services in remote settings of Nepal. Similarly, the National Health Education, Information and Communication Centre (NHEICC) started using cell phones to educate people on sexual and reproductive health. Additionally, there are many digital health information systems including HIV Surveillance, eTB register, mental health counselling in Nepal, being supported by World Health Organization (WHO), United Nations International Children's Educational Fund (UNICEF), and Save the Children (6, 16).

Though the internet service is accessible to less than 35% of the overall population in Nepal, the subscription of smartphones has increased from 0.043 per 100 people in the year 2000 to 139 per 100 people in 2020 (15). In a nutshell, various small-scale digital health programs are operational in Nepal but are often vertical in approach (6). Although digital health is not a silver bullet when offered as a stand-alone solution, deploying digital health intervention has successfully addressed public health issues in LMICs and can potentially do so in Nepal (17). There is an ongoing body of work in Nepal that has unearthed opportunities and challenges in rural digital health ranging from community health care centers to specialists in tertiary hospitals (18). All of them are based on specific health issues and carried out as pilot projects in specific regions of Nepal. For example, collaborative care for psychiatric patients in a rural setting (19), capacity building and text messaging intervention in the Dhanusha district (20), and dengue prevention through mobile SMS (21).

While the importance of digital health has been well acknowledged, it is imperative to understand the barriers and enablers for implementing digital health in Nepal that may guide the development and implementation of digital health intervention in Nepal. Therefore, this narrative review aims to provide a synopsis of Nepal's challenges and opportunities for implementing digital health initiatives.

## Methods

This review method was developed by following the guidelines and criteria set in Preferred Reporting Items for Systematic Review and Meta-Analyses (PRISMA) (22). The rapid review protocol was registered in PROSPERO (CRD42020199056).

### Search strategy

An electronic search was conducted between 1 January 2010 and 30 December 2021 through PUBMED, Scopus, CINAHL, and Google Scholar using the keywords: Telehealth, Telemedicine, e-health, digital health, challenges, implementation, and Nepal. These keywords were searched in Medline using the Boolean operators “AND” and “OR” (a detailed search strategy is provided in Box 1.

BOX 1 **List of search items**

**Challenges:** “Challenges” OR “Problems” OR “Difficulties” OR “Chance” OR “Possibilities” OR “Potential” OR “Scope” OR “Issues” OR “Concerns” OR “Obstacles” OR “Barriers.”
**Digital health:** “Digital health” OR “Telehealth” OR “Mobile health” OR “mHealth” OR “Telemedicine” OR “eHealth” OR “telehealth” OR “Remote medicine” OR “Teletherapy” OR Distance medicine.”
**Nepal** “Nepal” OR “Rural Nepal” OR “Urban Nepal” OR “Developing country.”

**Inclusion criteria:**

I. Primary studies focusing digital health in any setting in Nepal.
II. Primary studies regardless of study design (e.g., qualitative, quantitative, observational, pilot study, case study, and Randomised controlled trial (RCT)) were included in the study.

**Exclusion criteria:**

I. Studies on public health issues that did not consider digital health approach
II. Conference abstracts, commentaries, reviews, and letter to editor
III. Studies published in a language other than English.

### Study selection

Initial records retrieved from each of the four databases were imported into Mendeley, where duplicates were removed. On the title and abstract screening, studies were excluded based on the selection criteria. Two authors (RP and DB) did the abstract screening, whereas title screening was done by all three authors (RP, DB, and MKC). After those studies were subjected to full-text screening to determine their eligibility for inclusion, which was done independently and later discussed for confirmation in a group. Any reviewer discrepancies were resolved by conducting a group meeting and further discussing them with the supervisor (UNY).

**Figure 1:**
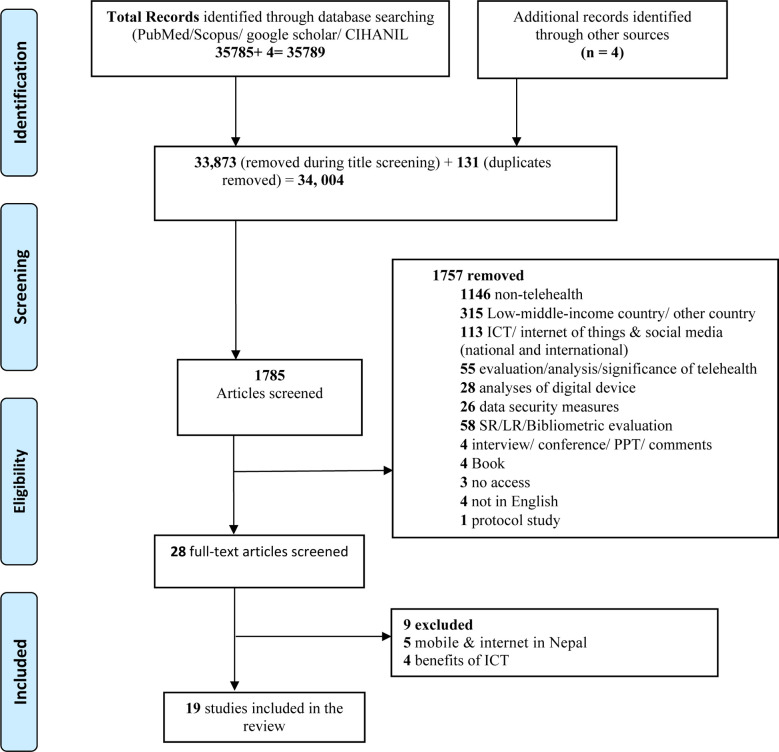
PRISMA 2009 Flow Diagram.

The PRISMA (22) diagram illustrates the screening process for the study selection (Figure 1).

### Data extraction

Data from the selected articles for final inclusion was divided among three reviewers (RP, DB, and MKC). The extracted data include the region, year, and authors of the publication. It also consists of the study design, service offered, the digital device/ technology used, the challenges faced, and the opportunities to deliver health services through digital devices /technologies. A data extraction sheet was developed (Table 1), and data were extracted from the information based on the selected 19 articles, and the results were discussed with the study team members.

**Table 1:** Data extraction sheet.

Data to be extracted	
Study outline	Reference details (title, authors, year)
	Research Question
	Digital Intervention
	Region
	Key findings
Study design and aim	Study design (qualitative, quantitative, Case studies, RCT / mixed methods)
	Aim of the study
Ethics	Discussion of ethical issues: No
Setting	Study area/setting
	Intervention delivery (community health workers/ Nurse/ GP/Hospital based
	Used technique/device
Representativeness	Number of participants included in analysis/ Target population

### Data synthesis

Data synthesis was done for both quantitative and qualitative data that highlighted the challenges and opportunities related to digital health interventions in Nepal. Both primary and secondary studies, including digital health interventions, were included in this review. Content analysis (23) was performed for descriptive evaluation of the selected articles, based on which specified outcomes were identified and arranged into two themes: challenges in implementation digital health, and future opportunities in digital health implementation.

## Results

The final literature search was conducted on 30 December 2021, which yielded 35,789 results through four different databases (CINAHL 28,046, Google Scholar 3650, Scopus 3884, and PubMed 209). After removing duplicates and screening the titles and abstracts of the articles using exclusion criteria, only 28 papers were selected for full-text screening. Of these 28 studies, 9 studies were excluded because of the following reasons: four mentioned the benefits of using ICT in Nepal, five were excluded for only focusing on the efficacy/ use of mobile phones and internet in Nepal. A total of 19 articles that met the eligibility criteria were included for data extraction. The characteristics and findings summary of all the selected 19 papers are mentioned in the Table 2, highlighting various challenges and future scope/ opportunities in implementing digital health services in Nepal.

**Table 2:** Data extraction on challenges and opportunities from the included studies.

Author	Intervention	Challenges	Benefit	Opportunities
Rai, 2013	**Type of study:** A case study. **Study area/ Setting**: Nangi village, Kathmandu, Model Hospital **Participants No./Target population**: 13 health workers and 96 patients. **Type of service offered:** dermatology, maternity, e-learning **Used technique/device:** video consultation **Intervention delivery through:** nurse, doctors, community health workers	– Staff turnover – Lack of IT skills – Lack of infrastructure – Uneven geographical distribution – No electricity/internet – Sometimes unavailability of specialist for teleconsultation – Lack of government support – Sometimes misdiagnosis	– Better diagnosis and treatment – Reduced child and maternal death – Increased patients concern for health care	– No extra cost – Better diagnosis and treatment – Telelearning by interns / other health workers
Morrison et al., 2013	**Type of study: Pilot study** **Study area/ Setting: Gulmi district** **Participants No./Target population: 24 health workers / 3 GP** **Type of service offered: phone consultation with the GP** **Used technique/device: landline phone/Mobile Phone** **Intervention delivery through: mid-level health workers.**	– Frequent power cuts – Network problem – Technical difficulties	– Easy consultation – Patients are more concerned towards health care.	– Stakeholders are positive
Piya, 2010	**Type of study**: A case study **Study area/ Setting**: OM hospital and research centre, Kathmandu, Nepal **Participants No./Target population**: 6 (out of 8 responded) health officers of OM Hospital. **Type of service offered:** not limited to single service/ consultations have been done for various types of illness Used technique/device: video consultation **Intervention delivery through:** Doctor and consultant	– Lack of IT knowledge of experienced doctors – Patients also need to know the usage technique – Sometimes, doctors are not willing to learn the new techniques – Sustainability (usually after execution of donor support, the program also gets terminated due to lack of resources) – Lack of availability of equipment – High installation cost – The poor and disturbing connection – Government support – Motivation	– Better health care facilities	– Low treatment cost for those who had to travel abroad for treatment. – Enhance technical skills among health professionals – Patient literacy about telehealth
Bhattarai et al., 2015	**Type of study**: A comparative-cross sectional **Study area/ Setting**: Manahari VDC and Hetauda, Makawanpur, Nepal. **Participants No./ Target population**: 40 people of age group 30 to 70 years (20 from intervention and 20 from non-intervention group) **Type of service offered:** Diabetic care **Used technique/ device:** tele-consultation **Intervention delivery through:** local doctors	N/A	– Reliable and better diabetic care – Management of diabetes care in rural area is feasible and comparable with urban areas.	– It can be used on a large scale in rural areas
Shrestha et al., 2018	**Type of study**: A case study **Study area/ Setting**: Semi-Urban Kathmandu, Nepal **Participants No./ Target population**: 2 patients infected with *Tinea Incognito* **Type of service offered:** Dermatology **Used technique/ device:** mobile phone **Intervention delivery through:** medical officer	N/A	– Successful treatment of long persistent skin problems	– Reliable, useful / cost-effective means of dermatological consultation
Ghimire et al., 2019	**Type of study: A cross-sectional study** **Study area/ Setting: Siddharthanagar Municipality, Bhairawa, Nepal** **Participants No./Target population: 100 participants of Siddharthanagar Municipality.** **Type of service offered: Tele stroke** **Used technique/ device: videoconferencing and image sharing technology** **Intervention delivery through: community people residing in Siddharthanagar Municipality, one member of one household who had heard about stroke was selected.**	– Technology level – More work on individuals – Installation time and cost for it. – Management issues include plasminogen activators’ effects. – Some individuals prefer to visit clinicians physically. – The concern of online data’ store and confident	– Benefits to stroke patients – Useful to physicians and the community regarding stroke education	– Improve diagnosis and treatment of acute stroke. – Useful in research of emerging stroke medications. – Enhance physicians and community education.
Cai et al., 2016	**Type of study: Prospective study** **Study area/ Setting: Department of plastic / reconstructive surgery at Kritipur Hospital Kathmandu, Nepal** **Participants No./ Target population: 17 individuals with healed burn scars** **Type of service offered: outpatient burn care** **Used technique/device: live video-conferencing** **Intervention delivery through: local occupational/physical therapist**	N/A	– Improve scar management	– Feasible – Sufficiently accurate for clinical assessment
Bhattarai et al., 2019	**Type of study**: Non-randomised quasi-experimental design **Study area/ Setting**: Ratnanagar Municipality (ward no.2) Chitwan District, Nepal **Participants No./ Target Population:** 300 participants from 50 households of Ratnanagar Municipality **Type of service offered:** Dengue prevention **Used technique/device:** Mobile Short Message Service (SMS) **Intervention delivery through:** District (Public) Health Officers, health workers and Female Community Health Volunteers (FCHV).	– Poor networks in geographically rural places.	– Can be taken as a tool for educating health against dengue and other diseases. – Improving preventing practices and behaviors regarding dengue in the community. – Perceived as enjoyable, informative, and trustworthy. – Acceptable and appropriate media.	– Can provide instant access to information to a large population – Effective measure devices in health knowledge and practices reduce transmission cases. – It can be considered a crucial public health advocacy tool to improve people health-related behavior.
Mandavia et al., 2018	**Type of study**: A cross-sectional **Study area/ Setting**: outpatient clinic of Sahodar Hospital, Lamjunj District, Nepal **Participants No./Target population**: 56 patients including adults and children **Type of service offered:** Otoscopy **Used technique/device:** Cupris device was used for ontological history and examination, and a recorded video was sent to the specialist. **Intervention delivery through:** trained non-medical workers	– May lead to less quality of the video and can impact results. – Requires more training and experiences	– It is a simple, quick, and valid tool for diagnosing ear-related diseases. – Based on the device result can plan of referral for further assessment. – Reliably screen and evaluation tool for ear diseases. – Simple and highly portable for images and history data capture rather than another tool (otoscope linked to a computer)	– Low cost, significantly less than the cost of an otoscope.
Hong et al., 2019	**Type of study**: Pilot study **Study area/ Setting**: Various District of Nepal (Ramechhap, Jiri, Dolakha / Charikot) **Participants No**./**Target population**: 346 participants from the four districts **Type of service offered:** Ophthalmology **Used technique/ device:** Paxos scope ophthalmic camera system attached to an iPod Touch 6th generation **Intervention delivery through:** ophthalmic technicians	– Uploading issues and lack of Wi-Fi access.	– Cost-effective, portable, hand-held design. – Durability, affordability, and their ability to take high-quality images.	– The opportunity to access health care
Swar et al., 2019	**Type of study:** Pilot study. **Study area/ Setting**: General Clinic, Accham Hospital **Participants No./Target population**: 300–400 patients per day **Type of service offered:** Mental health care **Used technique/ device:** videoconferencing **Intervention delivery through:** primary care providers (PCPs)	– Unreliability to electricity – Technical issues – Lack of trusting relationship between psychiatrists and patients due to remote communication or no face-to-face contact – A mismatch between psychiatric recommendations and the site’s capacity to implement them due to limited capacity	– Teleconsultation in rural parts of far western Nepal to improve the quality of mental health services – Solar back up/Gasoline generators for power cut problems	– Video-based training for primary care providers in mental health issues
Shrestha PL / Ellingsen, 2016	**Type of study:** Qualitative Case study. **Study area/ Setting**: Dhulikhel Hospital and 14 outreach clinics **Participants No./ Target population**: patients at 14 outreach clinics. **Type of service offered:** Dermatology, Gynecology **Used technique/ device:** Global System for Mobile Communications (GSM) based phone, Code Division Multiple Access (CDMA) phones, and Email. **Intervention delivery through:** paramedics	– Technical problems – High maintenance cost	– Management of dermatology, gynecology, and emergency cases through telemedicine in 14 outreach clinics – User friendly technology leads to a sustainable system	– Paramedics also learned through teleconsultation – Building relationships among paramedics, people, and specialists
Bhandari, 2016	**Type of study:** Cross- sectional study. **Study area/ Setting**: Sahid Gangalal National Heart Centre (SGNHC) **Participants No./Target population:** 80 cardiac patients from outpatient department who are 18 years and above. **Type of service offered:** tele-cardiac rehabilitation **Used technique/ device:** mobile-based telehealth **Intervention delivery through:** primary care physicians.	– Limitations in access to the Internet	– The substantial willingness of patients towards mobile-based telehealth/ use mobile calls for consultation/disease management.	– Promising measure to fill the gap in the existing health care and to increase the access of health care to rural people in Nepal – Opportunities to launch a large telehealth project with a target to cardiac and chronic patients requiring Continuous monitoring.
Mercado et al., 2017	**Type of study:** Pilot study. **Study area/ Setting**: Tilganga Institute of Ophthalmology (TIO) in Kathmandu, Nepal, Geta Eye Hospital and rural outreach centers **Participants No./Target population:** Patients at hospital and outreach clinics **Type of service offered:** mobile ocular imagining **Used technique/ device:** Paxos Scope smartphone camera adapter coupled with an iPhone 5. **Intervention delivery through:** two cornea specialists	– connectivity issues with use of digital software	Empowering community eye hospitals to relay information back and forth with tertiary eye centres, Tilaganga Eye hospital	Affordable, high-quality, mobile ocular imaging option for under-resourced parts of the world.
Bhatta, 2013	**Type of study:** Qualitative research method. **Study area/ Setting:** Rural Sindhupalchowk, Darchula district hospital and Patan Hospital. **Participants No./Target population:** Patients visiting Sindhupalchowk and Darchula district hospitals. **Type of service offered:** sexually transmitted diseases (STDs), HIV/AIDS, general medicine, podiatric, orthopedic, gynecology, dermatology and surgical cases **Used technique/ device:** video conference **Intervention delivery through** health workers of the respective hospitals	– Insufficient infrastructure resources and technology. – Difficult geographical distribution. – A weak policy of the government on rural-telemedicine programs. – Lack of funds and skilled manpower. – Frequent transfer of health workers.	– Underserved people from rural Nepal get an approach to health service. – Affordable and cost-effective health care service to countryside people. – Improve the health care system in rural Nepal.	– The government get an opportunity to look at health policy for future need. – Make to realize the challenges and limitations faced in providing health services in rural Nepal for improvement. – Connects rural districts hospital to tertiary level hospitals for medical support.
Adhikari et al., 2020	**Type of study:** Retrospective study design **Study area/ Setting:** Manekharkha and Bahunepati in rural municipality Banepa. **Participants No./Target population:** 15/ patients meeting eligibility criteria. **Type of service offered:** Tele physiotherapy **Used technique/ device:** mobile phone **Intervention delivery through:** local health assistant**s**	– They could not measure exercise adherence. – Barrier to accessing health care service in rural people.	– Participants feel a significant reduction in perceived pain due to various musculoskeletal conditions. – There is no significant difference between TPT and face-to-face physiotherapy.	– Telephone-based tele-therapy seemed a feasible option for pain management where high technology is beyond reach and has a literacy rate. – It could be a choice to deliver home-based rehabilitation to enhance the older population’s wellness.
Lama, 2011	**Type of study:** A Participatory Action Research method. **Study area/ Setting:** Dhulikhel Hospital and 3 remote health centres. **Participants No.**/**Target population:** Three group of participants; Doctors from Dhulikhel Hospital, Village health worker from Dhulikhel Hospital’s outreach and government health from from Dhulikhel health centers) **Type of service offered: Dermatology, Eye care** **Used technique/ device:** mobile phone and email **Intervention delivery through:** remote health care workers	– Participants refused to answer the questions and get involved in the discussion. – Difficult geographical distribution.	– People get supportive and affordable care services.	– Got the opportunity to observe and experience the dynamic changes in the knowledge, attitude, and practice of all the stakeholders. – Led to a completely different understanding of the difficulties between telemedicine interventions.
Meyers et al., 2017	**Type of study:** Pilot study **Study area/ Setting:** Rural Achham **Participants No./Target population:** 9 community health worker leaders (CHWL), 81 CHWs across nine village clusters serving 25,000 people. **Type of service offered:** data collection of the conditions like pregnancy and diarrhea. **Used technique/ device:** mobile phone **Intervention delivery through:** community health workers	– Staff turnover and technical difficulties. – Problem to access required telephone network. – Lack of training and inappropriate technical partner. – Poor process planning and management.	– Due to inadequate management and planning, the program failed to achieve its goal and collapsed.	– Provide information for future mHealth interventions in similar contexts. – Failure of the project makes them to realize what factors prevent them from achieving goal of m-Health management.
Basu et al., 2017	**Type of study:** A case study. **Study area/ Setting:** 2015 Earthquake affected areas of Nepal. **Participants No./Target population:** A large set of doctors for you (DFY) who exchanged messages from different earthquake relief operations. **Type of service offered:** supply of medical resources during different phases after the earthquake. **Used technique/ device:** Mobile phone **Intervention delivery through** medical experts (doctors)	– Information was only obtained and shared among DFY (Doctors for You) after a disaster. – The problem is in the transportation of medical personnel and supplies. – Electricity and save drinking water problems during that time.	– Real-time analysis of such online data helps to decision makers in forming resources and mapping strategies dynamically. – Provide medical aid and other needs to needy people during disaster crises.	– Provide a lesson that government should always be on standby to help in critical disasters.

As only a few studies reported, the enablers for implementing digital health are merged with the benefits and opportunities. The first enabler was that the stakeholders of rural areas were likely to adopt digital health services if supplied with good technical equipment (13, 24) and motivations (organizational and governmental support for digital health (programs) (24, 25). Second was the sustainable relationship between patients, paramedics and consultants which will further help sustain the digital health programs (19, 24, 26, 27).

### Challenges in the implementation of digital health

**Figure 2:**
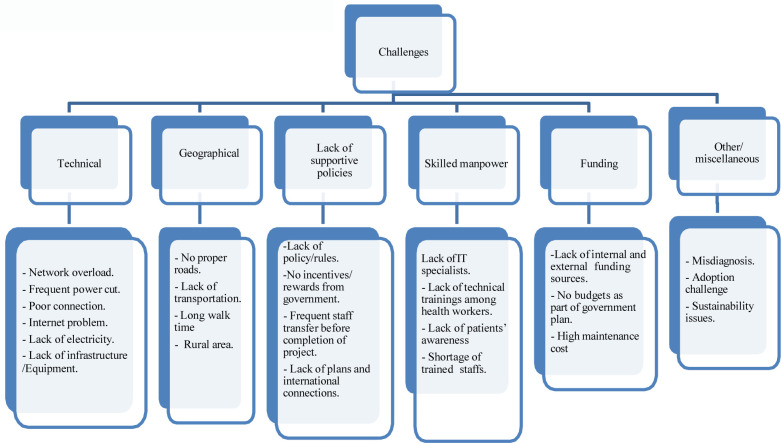
Challenges for implementing digital health interventions in Nepal.

In this context, challenges are defined as the problems or barriers faced by stakeholders (health care providers and patients) during the implementation of digital health programs in Nepal. Different challenges faced by stakeholders are categorized into six sub-themes: technical challenges, geographical challenges, policy challenges, lack of skilled workforce, funding challenges, and other challenges. All those themes and sub-themes were developed by analyzing various themes illustrated in Figure 2.

#### Technical challenges

Technical challenges are defined as technical issues faced during the implementation of telemedicine in Nepal. Technical challenges include network problems, frequent power cuts/ lack of electricity, blurred image, poor sound quality/cut- off the sound during video consultations due to slow internet connection, and interrupted service due to poor network quality. These technical issues were mentioned in 14 papers (10, 13, 19, 21, 24–26, 28–34), of which network connection problems (13, 21, 24, 31) and frequent power cuts (29, 31) were the major technical challenges for implementing digital health successfully.

### Geographical challenge

Five studies (10, 13, 24, 27, 32) mentioned rugged geography as one of the challenges faced in implementing digital health services in Nepal. The geographical factors identified by the authors were uneven geographical distribution like hilly and mountainous regions, which have made transportation difficult due to the lack of roadways in these areas. Also, the rough terrain has made the installation of mobile towers and other computer devices difficult and facing maintenance difficulties (21).

#### Lack of supportive policies

Lack of supportive policies was mentioned in four studies (10, 13, 24, 25) which were political instability, frequent transfer of trained doctors/ health workers, and lack of government support like lack of motivation by the government to their staff in terms of compensation and bonus salaries (24) have become a reason to end the telemedicine program.

#### Skilled workforce challenges

Five studies (10, 13, 24, 25, 28) mentioned the lack of skilled workforces, such as the lack of IT specialists, who could provide technical training to health workers about video consultations, and the lack of technical knowledge among health workers. Piya (24) also mentioned the lack of basic internet use and video consultations among health professionals. Furthermore, there was insufficiently trained staff at the local health care centers who could encourage the public towards digital health. Thus, the lack of specialists for teleconsultation has discouraged the program and interrupted the whole digital health project.

#### Funding challenges

Funding challenges in terms of buying costly equipment, high installation charges, and training staff were identified by four studies (10, 24, 26, 33). Digital health programs in Nepal depend on funding and volunteers; when funding stops, the whole project gets disturbed and terminated (24). Similarly, installing new and advanced technologies is expensive in a resource constrained country (26). Moreover, Basu et al. 2015 (32) mentioned the lack of IT specialists in Nepal, and it was financially challenging to recruit the experts from overseas.

#### Other challenges

Four studies mentioned that adaptation challenges and misdiagnosis are categorized as other challenges (13, 19, 24, 33). Adaption challenges make it difficult to train senior doctors who believe in face-to-face consultation rather than video consultations, and it was also challenge to convince patients about the benefits of digital health. Furthermore, one of the papers mentioned a case of misdiagnosis, which occurred through the conveyance of wrong messages due to an interrupted phone connection (13).

### Opportunities

**Figure 3:**
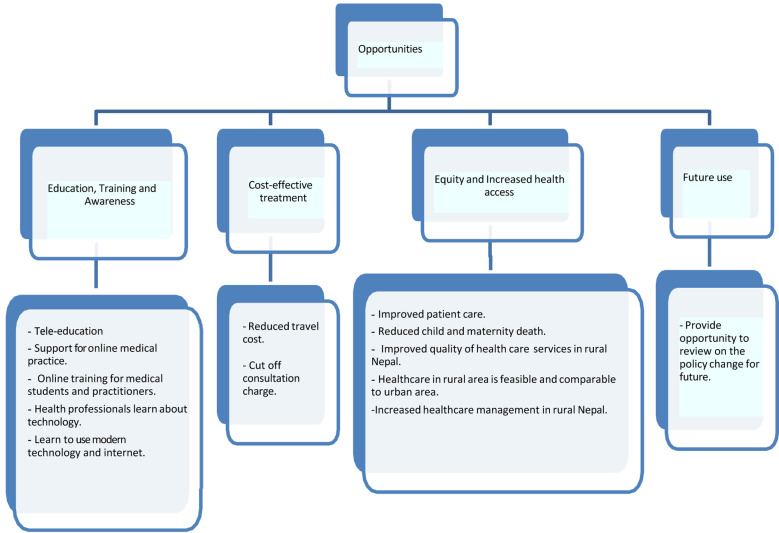
Opportunities for implementing digital health interventions in Nepal.

For digital health services, opportunities are defined as the service or facility provided by digital health to the public to deliver medical services regardless of distance and time through information and communication technologies. Opportunities are also categorized into four forms education, training and awareness; cost-effective treatment; equity and increased health access and future use (Figure 3).

In the context of digital health, opportunities are not just limited to using technology remotely to deliver health care services but also simultaneously adaptation by staff to the new working practices. Firstly, the professionals (health care providers) need to trust the digital system, reassure the patients of the privacy and confidentiality of collected health information, and ensure affordability for individuals and health care organizations (13).

Additionally, telehealth interventions' experiences and outcomes depend on the design details and factors like health literacy, digital literacy, and the quality of integration with clinical care pathways. To realize the long-term benefits of digital health, organizations need to collaborate and learn what, where, when, why and how it works well (24).

#### Education, training, and awareness

Digital health is a platform for all health stakeholders that provides an opportunity for health education, training, and awareness. Opportunities were mentioned in 10 studies- (13, 19, 21, 24–26, 32, 33, 35, 36) through different forms such as telesurgery and teleconsultation by medical students. It was mentioned that doctors learned about IT skills during health care delivery (13, 26), and patients also got the opportunity to learn about the use of technical devices (24).

#### Cost-effective Treatment

Cost-effective treatment was mentioned by four studies (10, 27, 29, 35). Cost-effective treatment in terms of reduced traveling costs with no excess consultation fee was mentioned in these articles. Patients from rural areas had to visit hospitals in the city for specialist consultations, which was reduced when they started getting digital consultations in their locality. As mentioned in a case study (37), untreated patients with face-to-face consultation were found to be adequately treated through mobile tele dermatology. Thus, patients were found satisfied and attracted to the quality of service they obtained through digital consultation, where they did not have to pay an extra for the specialist service.

#### Equity and Increased health access service

As mentioned in 12 studies (10, 13, 19, 21, 24, 26, 31–34, 36, 38), patients from rural areas were found to be more focused on their health issues and were interested in using digital health services. Similarly, another study by Sikhar Swar et al. (19) showed improvement in patients’ mental health status in far western regions. Digital health services delivered for diabetic care, childcare, and maternal health care in rural areas showed similar outcomes compared with urban health care facilities. Thus, telehealth service was more affordable and accessible to the public, and assistance was provided by the health professionals as required, which encouraged patients to use this service (38).

#### Future use

Future use of digital health was mentioned in four studies (10, 21, 34, 38) in different forms, like the opportunity to review digital health care policy and implementation on a large scale. Lots of successful pilot projects by Morrison et al. (31); Hong et al. (29), and case studies by Shrestha et al. (37) and Basu et al. (32), among few, have been conducted in various parts of the country yielding major positive outcomes with some drawbacks also. From all those outcomes, the government, including other stakeholders, can use the results for implementing those projects in revised form on a large scale. Doctors, nurses, and even patients need to be trained regarding digital health use and its translation into their workplace without any technical hassle (39).

## Discussion

This review explores the challenges of implementing digital health programs in Nepal before Covid 19 and future opportunities. The most recognized barriers were technical barriers such as network overload, frequent power cuts, lack of electricity, and internet problems (13, 19, 24, 29, 31, 34). Similarly, the lack of sufficient technology and infrastructure crisis (inadequate telephone network, Wi-Fi connectivity, and mobile phone penetration) for running digital health has also become a huge barrier to its implementation in Nepal (10, 21, 25). A study conducted in Bangladesh by Hoque et al. (39) found similar technical issues like lack of ICT infrastructures (lack of electricity and network problems), and a study done in India (40) mentioned the lack of broadband for quality of video consultation. Less than 85% of the houses in Nepal have access to electricity, which are facing rolling blackouts commonly known as load shedding for several hours and multiple times a day. Industrial production is also dependent on this limited power supply. Remote Nepal is mainly affected and has compelled the citizens to use alternate sources like small solar power systems or diesel for power supply (41). Some papers also found a lack of IT skills among doctors, nurses, and other health care workers. Younger doctors and interns are willing to learn about the new technology, but for senior doctors, it is hard to convince them to be tech-friendly (24). A similar pattern was also found in a study done by Hoque, M. R., Maximum, M. F. A., / Bao, Y. (39) among the older administrative staff who were found to be a bit resistant to adapting to new technology.

Apart from technical issues, a skilled workforce plays an important role in the smooth functioning of digital technology, but many lagging factors were found. On one side, there was a lack of operational skills among health care staff (10, 13, 25), and on another side, the literacy gap among people in the rural-terai region was found (20). Thus, training should be provided to the staff and awareness-raising initiatives among the patients (21, 28). A study from India (40) also emphasized the lack of technical training among paramedics. Lack of technical illiteracy in digital health intervention was also identified as a hindering factor among Pakistani citizens in a study by Ittefaq, M., / Iqbal, A (42).

Additionally, funding plays a vital role in supporting digital health services and, in the context of Nepal, is a major motivational factor for its sustainability. The WHO also has mentioned the lack of funding as a major factor for lagging digital health services in developing countries (43). Though the government has planned to expand the digital health facilities, a separate budget in this sector is not allocated, and does not fall into a top priority list. Thus, reimbursement can also be used as a motivational factor to support the volunteers/digital health in the long run. Studies from Bangladesh (39, 44) also found similar funding-related problems, including access to digital tools that affect the adaptation of digital health.

The new information and technology era may lead to new hope and more services for the public in Nepal, where geographically varied places and their landscape become barriers to health care delivery. Due to a lack of infrastructure and other facilities, people lag in accessing health services and have died from curable and preventable diseases. So, digital health and modern medical technology offer treatment, cure, and awareness in addressing health needs without traveling out of hometowns. Developing countries like Nepal, India, and Bangladesh and even developed countries like Australia have shown the cost-effective benefits of digital health in aged care centres and disability centres in remote areas (45).

Digital health interventions conducted in remote villages of Nepal have shown equal consultations and treatment benefits as received by urban patients (38). Hence, digital health empowering community hospitals and tertiary centers has shown fruitful application (25), thus extending to larger areas. Medical students were found learning through tele-education. The intern doctors of Kathmandu model hospital were virtually experiencing the surgery conducted in Korea through telesurgery. They were eager to learn about the techniques (13). In addition, mid-level health workers and volunteers also had telephone for video consultations to care for the patients (20, 31).

Furthermore, most of the digital health interventions we have included in our review were conducted in small areas or specific locations, with a positive outcome. This has the potential to expand digital health services in a broader context by empowering community health care centres through relaying information to and fro with the tertiary hospitals. For instance, a study carried out by Mercado et al., (30) at Tilganga Institute of Ophthalmology (TIO) in Kathmandu, and rural eye hospital in Dhangadi, and a rural cataract camp in Hetauda showed the potential of digital health further to expand their impact in other areas (30).

Digital health literacy is the most among doctors, and patients play an essential role while implementing digital health. Few papers (13, 21, 24) have mentioned the importance of digital literacy among the stakeholders. The confidentiality of patients’ information is also a significant concern. Morrison et al. noted that confidentiality might not be considered an essential factor for telephone-related consultations (31). Pradhan et al. mentioned that Nepal has no specific data protection law (46). Additionally, the negligence in patients’ data protection systems while conducting digital health programs was mentioned by Rai (13).

A quantitative study shows equal access to diabetes care in rural and urban areas. Teleophthalmology also has reduced the travel cost (29, 38). Patients were eager about digital health (34) and were found equally satisfied whet her they were treated face to face or through tele ophthalmic means (29). Additionally, the majority of the patients’ were comfortable with telephone consultation (34). Phone-based SMS was also used as a health promotion tool for dengue control and increased awareness about nutrition (21).

Nepal's government should take heed to developing a national digital health strategy that may guide the effective implementation of digital health interventions in Nepal. Evidence has highlighted the importance of digital health education among health care providers (34). Moreover, a center for digital capacities and knowledge should be established to train, coach, and facilitate the human resource to digitalize the health system (46), which motivates them to uptake new technology without hesitation (34). It may allow the government to deliver services in a people-centered approach improving digital health literacy and the quality and safety of health services, thereby helping the country to achieve universal health coverage.

### Strength and Limitations of the study

Like other studies, this paper does have strengths and limitations. The strengths include: (i) findings based on the review of peer- reviewed journal articles, and (ii) findings might guide the policy and practice for digital interventions in Nepal. This study had some key limitations. First, the literature search was done in selected databases using key search terms which might not have captured all the published evidence. Secondly, the study only reviewed articles published within a particular time framework and did not include grey literature; therefore, findings should be interpreted carefully. Thirdly, the search was limited to English, meaning this study did not consider articles published in Nepali.

## Conclusion

The study identified various challenges and opportunities that can guide the development and successful implementation of digital health interventions in Nepal. Moreover, decision-makers should involve wider stakeholders including information technology experts and the developmental partners in building the capacity of public health facilities, and workforces to effectively tailor and deliver digital health interventions in Nepal. While digital transformations have great potential to benefit population health, they may exacerbate social inequalities. Therefore, future research should focus on social and cultural determinants of digital health literacy at the professional level (those who develop, deploy, recommend, and prescribe digital health services) and community level (those who use digital health services). This may guide the development of people-centred digital health information that can address the practical, comprehensive needs of the people.

## Data Availability

The original contributions presented in the study are included in the article/Supplementary Material. Further inquiries can be directed to the corresponding author/s.
